# Body temperature at nursery admission in a cohort of healthy newborn infants: results from an observational cross-sectional study

**DOI:** 10.1186/s13052-020-0810-z

**Published:** 2020-04-15

**Authors:** Daniele Merazzi, Ilia Bresesti, Paolo Tagliabue, Maria Grazia Valsecchi, Paola De Lorenzo, Gianluca Lista, Collaboration Group, Irene Daniele, Rosanna Restelli, Lina Bollani, Antonella Maini, Cristina Bellan, Emanuela Zappella, Lorenzo Colombo, Gabriele Sorrentino, Giovanni Ciraci, Marcella Lomazzi, Ilaria Lombardo, Marilena Ferraresi, Luciana Pagani, Luca Bernardo, Manuela Romani, Massimo Agosti, Stefano Martinelli, Giuseppe Miraglia, Emilio Palumbo, Veronica Menghi, Claudio Cavalli, Arianna Paternieri, Daniela Messina, Maddalena Teani, Roberta Barachetti, Franca Lazzari

**Affiliations:** 1 Division of Neonatology, “Valduce” Hospital, Como, Italy; 2 Division of Neonatology, “V. Buzzi” Children’s Hospital, ASST-FBF-Sacco, Milan, Italy; 3 Division of Pediatrics, “L. Sacco” Hospital, ASST-FBF-Sacco, Milan, Italy; 4 Division of Neonatology, “S. Gerardo” Hospital, MBBM Foundation, Monza, Italy; 5 Center of Biostatistics for Clinical Epidemiology, School of Medicine and Surgery, University of Milano-Bicocca, Monza, Italy; 6 Center of Biostatistics for Clinical Epidemiology and Pediatric Clinic, School of Medicine and Surgery, University of Milano-Bicocca, Monza, Italy

**Keywords:** Newborn, Thermoregulation, Delivery room

## Abstract

**Background:**

Exposure to hypothermia is somehow unavoidable when a baby comes to life. This is the reason why any possible effort should be made by every caregiver involved during birth, from labour to transfer into the maternity ward, to reduce it. Hypothermia has widely shown to be related to several neonatal problems, and the risks are more relevant when the babies are born prematurely.

**Method:**

An observational study was conducted in April 2016 to assess the current practises to avoid hypothermia at birth in 20 Italian neonatal units. Each unit introduced local improvements in clinical practice and the same observational study was repeated 1 year later.

**Results:**

A total of 4722 babies were analysed. An overall increase in adherence to local and international recommendations emerged from our study. Significant differences between 2016 and 2017 were found in regard to neonatal temperature at nursery entry (36.3 °C vs 36.5 °C, respectively, *p* < 0.0001), delayed cord clamping practice > 60″ (48.1% vs 68.1%, respectively, *p* < 0.0001) and skin-to-skin practice > 60′ (56.3% vs 60.9, respectively, *p* = 0.03). Statistical correlations with the risk of hypothermia were found for delivery room (OR 0.88 (CI 95%0.83–0.94), *p* < 0.0001) and maternal temperature (OR 0.57 (CI 95% 0.48–0.67), *p* < 0.0001).

**Conclusion:**

Periodical assessment of the delivery room practice has shown to be effective in improving adherence to the international recommendations. Relationship between neonatal hypothermia and several other variables including the delivery room and mother temperature underlines how neonatal thermoregulation starts immediately after birth. Hence, a multi-disciplinary approach is needed to provide the optimal environment for a safe birth.

## Background

Adaptation to extra-uterine life is a complex process, including a rapid and sudden change in thermal homeostasis of the baby. Once they leave maternal milieu, in fact, all babies are exposed to an unequivocal tendency towards hypothermia, which is inversely proportional to their gestational age (GA). Maintenance of thermal homeostasis is crucial for the success of postnatal transition and its imbalances have been reported to be responsible for glycaemic disorders, respiratory distress and increased mortality [1, 2]. Thus, control of the body temperature needs to be managed carefully by the attending caregivers at birth.

Thermal care practices have long been a milestone of neonatal care, and temperature maintenance for newborns has evolved over the years, including the use of different devices (such as radiant warmers and heated mattresses) and family-led practices (such as delayed bathing, wrapping of the neonate, hats use and skin-to-skin care) [3]. Delivery room (DR) temperature as well plays an important role in the thermal homeostasis.

Both hypothermia and hyperthermia should be avoided during stabilisation and upon admission to the neonatal unit, and for this reason, systematic monitoring of temperature (preferably skin and rectal) is therefore advisable to prevent inappropriate uncontrolled temperature variations.

The ideal range for newborn body temperature is not unequivocally defined, but there is consensus on a target range between 36.5 °C and 37.5° [4, 5].

In clinical practice, this goal is not routinely achieved, due to either scarce adherence to the international recommendations or to the different procedures adopted by caregivers.

To investigate the current clinical practice, an observational study was carried out in 20 neonatal units in Lombardy (Italy), enrolling 2376 newborn infants born in April 2016. Data were collected through an ad hoc questionnaire and were fully anonymized.

This study revealed unexpected findings on thermoregulation in the DR. Although neither significant adverse events nor increased morbidity were recorded, infants at nursery entry showed a median body temperature below the suggested range (36.3 °C, ranging from 35.7 °C–36.9 °C). Also, differences between centres were detected regarding the management after birth. These findings were presented through an oral presentation during the annual regional meeting of Italian Neonatology Society in 2017 [6]. Then, a stricter adherence to the “warm chain” of the World Health Organization (WHO) recommendations [5] was advocated. Every unit was then responsible for the implementation of its own local policies to increase adherence to international recommendations.

We here report the data of the same observational study performed in 2017 in the same 20 units.

Our primary aim was to compare practices of the neonatal management after birth in 2016 and 2017.

In addition, we aimed to assess which factors were associated with neonatal temperature at and immediately after birth. For this purpose, with the 2017 cohort we extended data collection to maternal and DR temperature at the time of delivery.

## Methods

In April 2017, we conducted an observational, cross-sectional, non-interventional study among 20 Italian neonatal units in Lombardy.

We collected data of inborn healthy term or late preterm infants (≥ 35 weeks’ GA) during the immediate post-partum period and before the nursery room entry. All the data were collected through an ad-hoc questionnaire.

Hypothermia was defined according to the WHO as temperature < 36.5 °C and was classified into mild (36–36.5 °C), moderate (32–35.9 °C) and severe (< 32 °C) [5].

Body temperature (skin or rectal according to local protocols), peripheral oxygen saturation and heart rate were recorded 2–3 h after birth. Data on the following potential risk factor for hypothermia were collected: mode of delivery, maternal analgesia during delivery, cord clamping (CC) modality, skin-to-skin contact (StS), bathing during the first 2 h of life, maternal body temperature at birth and DR temperature.

Parental consent was waived because of the observational and non-interventional nature of the study. The data were handled according to regulations of data protection agency in our country. After being fully-anonymised, data were locked and stored for statistical analysis and archived in accordance with the GCP guidelines.

### Statistical analysis

Differences between the 2016 and 2017 cohorts in terms of infants’ characteristics at birth and nursery entry. The incidence of risk factors for infants’ hypothermia were assessed with the Fisher exact test (for categorical variables) and the Wilcoxon rank-sum tests (for continuous variables). The impact of the potential risk factors on the occurrence of hypothermia was analysed with the logistic regression model and expressed in terms of odds ratio and 95% confidence intervals. All tests were two-sided. Analyses were performed with SAS 9.2.

## Results

We screened a total of 2346 newborn infants born in April 2017 at 20 neonatal units of any level of care in Lombardy (Italy). Infants’ characteristics at birth and nursery entry and potential risk factors for neonatal hypothermia are described in Table 1 and compared with the 2016 cohort of 2376 neonates. Infants’ characteristics at birth were similar in the two cohorts, while several characteristics at nursery entry were significantly different. In the 2017 cohort, preterm infants (< 37 weeks GA) were 101 (4.3%) and, among these, 35 neonates were < 36 weeks GA (1.5%). Infants with birth weight < 2500 g were 110 (4.7%). The incidence of hypothermia after birth was significantly higher in 2016 as compared to 2017 (59.6% vs 48.4% respectively, *p* < 0.0001). The median body temperature at nursery entry was significantly lower in 2016 cohort in respect to 2017 cohort (36.3 °C vs 36.5 °C respectively, *p* < 0.0001). Practice of DCC > 60″ was significantly more common in 2017 as compared to 2016 (69.1% vs 48.1% respectively, *p* < 0.0001). The StS > 60′ duration was more practised in 2017 cohort (60.9% vs 56.3%, *p* = 0.03).

**Table 1:** Infants’ characteristics at birth, at nursery entry and potential risk factors for hypothermia in the 2016 and 2017 cohorts. Statistical significance was defined as *p* < 0.05

	Survey 2016 *N* = 2376	Survey 2017 *N* = 2346	*p*-value
**Infants’ characteristics at birth**			
Gestational age (weeks)			
Infants with available data	2369	2307	
median (range)	39.3 (33.3–42.0)	39.3 (34.0–42.0)	0.41
Birth weight (g)			
Infants with available data	2369	2346	
median (range)	3290 (1900–5000)	3290 (1780–5130)	0.53
Gender (male)			
Infants with available data	2376	2346	
N (%)	1230 (51.8%)	1226 (52.3)	0.75
**Infants’ characteristics at nursery entry**			
Neonatal hypothermia (< 36.5 °C)			
Infants with available data	2376	2346	
N (%)	1415 (59.6)	1135 (48.4)	< 0.0001
Temperature at nursey entry			
Infants with available data	2376	2346	
median (range)	36.3 (34.2–37.7)	36.5 (34.0–38.1)	< 0.0001
Heart rate at nursery entry			
Infants with available data	1983	1871	
median (range)	133 (80–186)	134 (85–199)	0.13
SpO_2_ at nursery entry			
Infants with available data	1984	2006	
median (range)	99 (85–100)	99 (80–146)	0.09
**Risk factors for infants’ hypothermia**			
Vaginal delivery			
Infants with available data	2375	2344	
N (%)	1820 (76.6)	1802 (76.9)	0.86
Maternal analgesia administration			
Infants with available data	2340	2320	
N (%)	963 (41.2)	846 (36.5)	0.001
Delayed cord clamping (> 60″)			
Infants with available data	2277	2127	
N (%)	1094 (48.1)	1469 (69.1)	< 0.0001
Skin to skin contact			
Infants with available data	2369	2345	
N (%)	1610 (68.0)	1523 (64.9)	0.03
Duration of skin to skin > 60′			
Infants with available data	1610	1523	
N (%)	907 (56.3)	927 (60.9)	0.03
Bathing after birth (before temperature recording)			
Infants with available data	2314	2346	
N (%)	1123 (48.5)	1347 (57.4)	< 0.0001
Maternal temperature			
Infants with available data	–	2160	
median (range)	–	36.5 (34.7–39.1)	–
Room temperature			
Infants with available data	–	1716	
median (range)	–	24.5 (19.0–30.0)	–

Table 2 describes the analyses on the potential risk factors of neonatal hypothermia in the 2017 cohort. According to the univariate analyses, the following factors had a significant impact on the risk of hypothermia: type of delivery (caesarean section), analgesia during delivery, duration of StS < 60″, maternal body temperature < 36.5 °C, and DR temperature < 25 °C. Delayed CC > 60″ had a borderline, non-significant impact. When these risk factors were jointly evaluated with a multivariable logistic regression model all, except the type of delivery, confirmed their statistically significant role in the occurrence of hypothermia.

**Table 2:** Results of the univariate and multivariable logistic models on the risk of hypothermia

Risk factor for hypothermia	Univariate Analyses		Multivariable Analysis	
	OR (95% CI)	*p*-value	OR (95% CI)	*p*-value
Type of delivery (cesarean vs vaginal)	1.70 (1.40–2.06)	< 0.0001	1.09 (0.79–1.50)	0.62
Analgesia administration (yes vs no)	1.49 (1.26–1.76)	< 0.0001	1.58 (1.25–2.01)	0.0002
Delayed cord clamping (> 60″ vs < 60″)	1.19 (0.99–1.43)	0.06	1.69 (1.31–2.19)	< 0.0001
Duration of skin to skin (> 60′ vs < 60′)	0.71 (0.60–0.84)	< 0.0001	0.54 (0.43–0.68)	< 0.0001
Bathing after birth	0.91 (0.77–1.07)	0.26	–	–
Infant warmer	0.94 (0.79–1.13)	0.52	–	–
Maternal temperature	0.57 (0.48–0.67)	< 0.0001	0.54 (0.43–0.67)	< 0.0001
Room temperature	0.88 (0.83–0.94)	< 0.0001	0.85 (0.79–0.92)	< 0.0001

*OR* odds ratio, *CI* confidence interval

We also evaluated baseline characteristics at birth, but none showed a significant impact on hypothermia and were thus excluded from further analyses: female gender (OR 0.97 CI95%, 0.83–1.14, *p* = 0.73), birth weight < 2500 g (OR 1.25 CI95%, 0.85–1.83, *p* = 0.26) and GA < 37 weeks (OR 1.30 CI95%, 0.87–1.94, *p* = 0.20).

We also compared maternal and DR temperature in neonates with and without hypothermia: mother of neonates with hypothermia had significantly lower body temperature than that of normothermic neonates (median [range] of 36.4 (35.0–38.8) vs 36.6 (34.7–39.1), *p* < 0.0001). The temperature of DR for babies with hypothermia was 24 °C (range 19–28 °C), significantly lower than that measured in DR where normothermic babies were born, 24.9 °C (range 19–30 °C, *p* = 0.0003).

No clinical adverse events was reported in either cohort and the lowest neonatal temperature (34.3 °C) was associated with water birth.

## Discussion

Our data describe the clinical management of temperature control in the DR in a representative cohort of infants born in Lombardy (Italy) in April 2017, after local implementations in clinical practice were introduced. Significant differences have been found regarding the incidence of hypothermia after birth and regarding the proper duration of DCC and StS. Maternal temperature at birth and DR temperature have also shown correlation with neonatal temperature after birth.

An important goal for all the caregivers attending the DR is to provide the best setting for both mother and her baby in term of comfort and safety. Since the influence of body temperature on newly born infants have been widely investigated and has shown to be related to several adverse outcomes [1, 2], great commitment should be dedicated to minimising heat loss [7, 8].

Our data showed that the median neonatal body temperature when admitted to the nursery was slightly lower than that suggested by international recommendations [5, 9]. However, even if still nearly half of our babies developed hypothermia after birth, severe hypothermia was not detected in any of them. Moreover, the ideal temperature target range was maintained in a significantly higher number of infants as compared to 2016.

Our cohorts included mainly healthy term and only a few late preterm infants (4.3%), and this could explain why no adverse events were reported. In fact, moderate (32–34 weeks’ GA) and especially very (28–32 weeks’ GA) and extremely preterm infants (< 28 week’s GA) are indeed at much higher risks for complications when exposed to hypothermia, though mild and brief as it is in the period between birth and maternity ward entry. This aspect warrants to be underlined to sensitise all the caregivers involved from labour until birth.

Available evidence shows that StS is one of the most effective procedures for homeostasis maintenance in the newborns [2, 3]. Bonding is usually performed immediately after birth on the mother’s chest, and it is more frequent after vaginal delivery than after caesarean section [10]. In vaginally delivered babies, the positive impact of bonding on neonatal thermoregulation have been widely demonstrated, both in term and preterm infants [11], while there is still controversial evidence regarding the role of StS after caesarean section [12, 13]. Neonatal units perform StS with different modalities (eg duration), and there is ongoing debate about the impact of its duration on neonatal physiology [14]. However, bonding is recommended for at least 60 min, especially with preterm infant, as this timing also allows a complete sleep cycle [15, 16].

Our data showed that, although overall StS practice decreased significantly after 1 year, in 2017 StS contact > 60 min was performed in about 2/3 of the babies and increased significantly as compared to 2016. However, again we must consider that our cohorts included mainly term or late preterm infants, born by vaginal delivery in most cases, where immediate clinical interventions are rarely needed. We have no data on the reasons why the StS contact was either not performed or interrupted earlier than recommended. It would be interesting to further explore this aspect for a better understanding of the limitations encountered by operators. In our cohort, the risk of hypothermia was significantly reduced when StS was performed for > 60 min. This interesting finding suggests that, in StS, “duration matters”. For this reason, we believe that there is still room for improvement, in order to make StS of appropriate duration a perinatal standard of care.

We also evaluated the impact of DCC on hypothermia. Its benefits have been shown by several randomized trials, and include increased placental transfusion, cardiovascular stability, cerebral oxygenation and lower risk for both severe intraventricular haemorrhage and late-onset sepsis [17]. The higher duration of DCC (60–75 s) has also been associated with a lower incidence of hypothermia at birth in preterm infants [17]. Our data showed a significantly increased use of DCC > 60 s duration in 2017 as compared to 2016. This change in the DDC policy may also be due to the publication of recommendations edited by the Italian Neonatology Society and shared with the Italian Obstetrics and Gynecology Society [18]. Surprisingly, no significant difference was found between babies underwent DCC > 60″ and those who did not in term of hypothermia. Moreover, in our study DCC > 60″ seemed to be related to increased risk for hypothermia. These controversial results warrant further investigation and highlight the importance of an accurate management of the baby while DCC is performed, in order to avoid massive heating loss.

In the 2017 study, we extended data collection to maternal and DR temperature, since we hypothesized that maternal temperature could influence the maintenance of baby’s body temperature and also that there could be an interaction between DR temperature and mother-infant’s homeostasis. During prenatal life, fetal temperature is about 0.5 °C higher than maternal one and variations in maternal temperatures affect the fetus. At birth, the baby undergoes a sudden change in the surrounding temperature. Thus, several approaches are advisable to limit this thermal shock. Our data showed that maternal temperature significantly affected that of the neonate, However, mothers of hypothermic babies had a median temperature of just 0.2 °C lower than those of normotermic neonates. Of note, according to our results, 1 °C increase in maternal temperature is associated with about 40% reduction of risk for neonatal hypothermia. This interesting finding highlighted how even apparently minimal difference can have a major clinical impact on neonates. The control of maternal temperature in the DR is challenging for obstetricians, especially when caesarean section is performed. In fact, women who underwent this surgery frequently experience a decrease in temperature, which often remains undetected [19]. Operating theatres are kept cold for surgical reasons, but this negatively affect thermoregulation of the mother, which in turn influence the neonate’s temperature. Especially when spinal morphine is used, the intra-thecal maternal hypothermia is more relevant and the temperature is about 0.5–1 °C lower [20, 21]. We can speculate that it might contribute in reducing the efficacy of StS in thermoregulation of the newborn. Interestingly, in our study we found that both maternal analgesia and caesarean delivery were relevant risk factors for neonatal hypothermia when evaluated separately. However, when included in a joint model with the other risk factors, only analgesia confirmed its impact on the risk of hypothermia. These findings further confirm the deep interaction between external variables and maternal factors in neonatal thermal homeostasis and the need for a multi-disciplinary commitment to compensate the negative effects of unavoidable interventions.

Delivery room warming seems to be one of the most useful intervention to maintain both maternal and neonatal normothermia. As shown by Horn and colleagues, the use of passive insulation only (e.g. blankets) is less effective than active forced-air warming [22]. The European Resuscitation Council (ERC) recommends a DR temperature of 26 °C, similarly to the WHO, which suggests 25 °C [5, 9]. Our data reflect a scarce adherence to both recommendations, since the median temperature was 24.5 °C. Noteworthy, while for babies with normothermia the median DR temperature was close to the recommended one (24.9 °C), for those with hypothermia DR had a significantly lower temperature (24 °C, *p* = 0.0003). Moreover, the increase of 1 °C in the DR temperature led to about 10% reduction of the risk of neonatal hypothermia. These results support the recommendation to avoid temperature below 25 °C.

Our data, however, should be interpreted with caution, since this study has some limitations.

First, we cannot provide detailed descriptions of the local policies implemented in each unit after 2016 since they were not standardized between all the participants. These preclude us from drawing broad conclusion on how to implement effectively neonatal practices immediately after birth.

We did not differentiate between tertiary and primary centers. This might have affected our findings, since it is reasonable to assume that there are slight differences in the DR management between centers of different levels of care. In addition, logistics may quite vary between centers, thus temperature measurement could have been markedly affected, biasing the interpretation of the results. Also, we did not perform a detailed analysis of the effects of GA on the incidence of hypothermia, given the lack of very premature infants in our cohorts and the paucity of late preterm. All these aspects warrant further investigations.

## Conclusion

Given the extreme vulnerability to body temperature decrease of newly born infants, it is crucial to provide the optimal thermoregulation from the DR. Our study suggests that periodic audits of the current practice are effective in identifying and improving critical aspects of DR management that may result in neonatal hypothermia. In addition, it confirms that several practices at birth play a role in reducing the risk of neonatal hypothermia; in particular, even moderate changes in the DR and in the maternal body temperature can affect the baby’s thermal homeostasis.

## Data Availability

All the data are available upon reasonable request to the corresponding author.

## Abbreviations

DDC: Delayed cord clamping
DR: Delivery room
GA: Gestational age
StS: Skin to skin
